# bmlTUX: Design and Control of Experiments in Virtual Reality and Beyond

**DOI:** 10.1177/2041669520938400

**Published:** 2020-07-17

**Authors:** Adam O. Bebko, Nikolaus F. Troje

**Affiliations:** Department of Biology and Centre for Vision Research, York University, Toronto, Ontario, Canada

**Keywords:** 3D perception, perception, virtual reality, stereopsis, perception/action, multisensory/cross-modal processing

## Abstract

Advances in virtual reality technology have made it a valuable new tool for vision and perception researchers. Coding virtual reality experiments from scratch can be difficult and time-consuming, so researchers rely on software such as Unity game engine to create and edit virtual scenes. However, Unity lacks built-in tools for controlling experiments. Existing third-party add-ins requires complicated scripts to define experiments. This can be difficult and requires advanced coding knowledge, especially for multifactorial experimental designs. In this article, we describe a new free and open-source tool called the BiomotionLab Toolkit for Unity Experiments (bmlTUX) that provides a simple interface for controlling experiments in Unity. In contrast to existing tools, bmlTUX provides a graphical interface to automatically handle combinatorics, counterbalancing, randomization, mixed designs, and blocking of trial order. The toolbox works *out-of-the-box* since simple experiments can be created with almost no coding. Furthermore, multiple design configurations can be swapped with a drag-and-drop interface allowing researchers to test new configurations iteratively while maintaining the ability to easily revert to previous configurations. Despite its simplicity, bmlTUX remains highly flexible and customizable, catering to coding novices and experts alike.

## Introduction

Recent advances in virtual reality (VR) technology have been driven by a large consumer-oriented market that aims at the development of a new medium for computer games. Head-mounted displays have become affordable, lightweight and user-friendly, screen refresh rates and spatial resolution have improved tremendously, and new tracking technologies reduce lag between head motion and display update below perceptual thresholds (Scarfe & Glennerster, 2019).

Not surprisingly, vision research is quickly adopting this new technology (de Gelder et al., 2018). In contrast to existing display technologies, VR offers a critical new depth cue, active motion parallax, that provides the observer with a location in the virtual scene that behaves like true locations do: It changes in predictable ways as the observer moves (Troje, 2019; Wexler & Van Boxtel, 2005). The contingency between observer motion and the resulting change in visual stimulation is critical and technically challenging as it requires an efficient, lag-free integration of measurements of head position and orientation with real-time stimulus generation.

As with hardware, the software that implements these tasks is geared toward the entertainment industry. Game engines such as Unity and Unreal Engine are widely used for game development. They support development of normal screen-based computer games using both 2D and 3D graphics, but their main strength lies in their ability to control head-mounted displays and provide simple tools to render highly detailed and realistic graphics. In that function, they are also becoming increasingly adopted by vision researchers.

Both game engines have similar functionality but also unique strengths and weaknesses. Unity is generally more approachable for users without software engineering backgrounds and provides a large quantity of free high-quality online tutorials. For this reason, it seems to be somewhat more popular among research laboratories than Unreal Engine.

Experiments in vision science are often structured using a factorial design, in which several independent variables are systematically varied, while participant responses are recorded in dependent variables. Participants then go through a series of trials, in which they are presented with different manipulations encoded in the values of one or many independent variables. Factorial designs typically involve some form of counterbalancing wherein enough trials are presented so that participants are exposed to all desired manipulations. Experimenters sometimes create blocks of trials that group trials based on the value of one or more independent variables. In addition, researchers often randomize the order of trials or counterbalance the order of blocks to control for order effects.

Unity does not provide a straightforward way to implement factorial structures. Coding experiments from scratch is time-consuming and difficult especially for studies using VR (de la Rosa & Breidt, 2018). In this article, we describe a new Unity plugin called the Biomotion Lab Toolkit for Unity Experiments (bmlTUX) that provides a flexible interface with Unity to simplify the creation of experiments and reduce the amount of coding required. The toolkit supplies the majority of repetitive code that is required for running most experiments, thereby enabling experiments to be created in Unity with minimal coding.

Two other Unity plugins have recently been published that simplify the process of creating experiments in Unity. The first major tool that was recently released is the Unity Experimental Framework (UXF; Brookes et al., 2019), which provides a backend platform that greatly simplifies the process of running experiments. This framework handles the code to run trials in the order that the experimenter has specified. However, setting up a simple experiment is not trivial and requires a user-provided text file containing a list of variables and their values. Critically, the experimenter must write complicated code to convert this text file into a sequence of trials including writing code to determine trial order, counterbalancing, randomization, blocking, and so on.

The second main tool is the Unified Suite for Experiments (USE; Watson et al., 2018) which provides more flexibility and control over experiment flow compared with UXF. The USE framework is based on a custom engine that can interface with external equipment that synchronizes high-precision timing and measurement. USE also provides easier integration with peripherals and hardware such as digital-analog converters that can interface with brain imaging systems or electrophysiological recording devices. The extra power and flexibility come at the cost of being more difficult to set up compared with UXF, even for very simple experiments. Like UXF, USE also requires a user-provided text file and code that specifies the exact sequence of trials.

Both existing tools greatly simplify the process of running experiments in Unity, but they do not aid in experimental design and do not provide the combinatorics of a factorial approach. All related functionality must be coded manually by the experimenter (i.e., variable entry, trial order, counterbalancing, randomization, and blocking). This process requires a strong background in coding and can be very time-consuming even for the simplest of experiments. Therefore, changes to the design usually require large changes to the associated code. Difficulty in initial setup and subsequent modification of experiments make testing new ideas and experiments much more difficult and slow down the process of iteration and refinement of experimental design. bmlTUX fills this gap in functionality by providing tools to aid in *both* the design and the execution of experiments in Unity. The toolkit is designed to simplify the setup and reduce the required code needed to get experiments up and running quickly. Unlike the above tools, our system can automatically handle the difficult task of specifying a mixed factorial design with multiple, potentially nested factors and turning it into an executable experiment. It provides the combinatorics of both random and counterbalanced factors, manages mixed designs with both within-subject and between-subject factors, and takes care of blocking and randomization of trial order. This functionality thus removes one of the most difficult aspects of creating experiments. In addition, the toolbox works *out-of-the-box*, meaning that a very simple experiment can be created with less than 20 lines of code (described and shown later). Using more advanced scripting features, the toolkit can be customized indefinitely to provide further custom functionality.

The toolkit is also designed to facilitate creating experiments incrementally. Variables and manipulations can be added or removed without requiring any changes to an experiment’s code, allowing for fast changes to test out new functionality. Furthermore, a single experiment can save multiple design configurations that can be swapped with a drag-and-drop interface. Therefore, researchers can test new configurations iteratively while maintaining the ability to easily revert to previous configurations if needed.

## Overview

**Figure 1.**
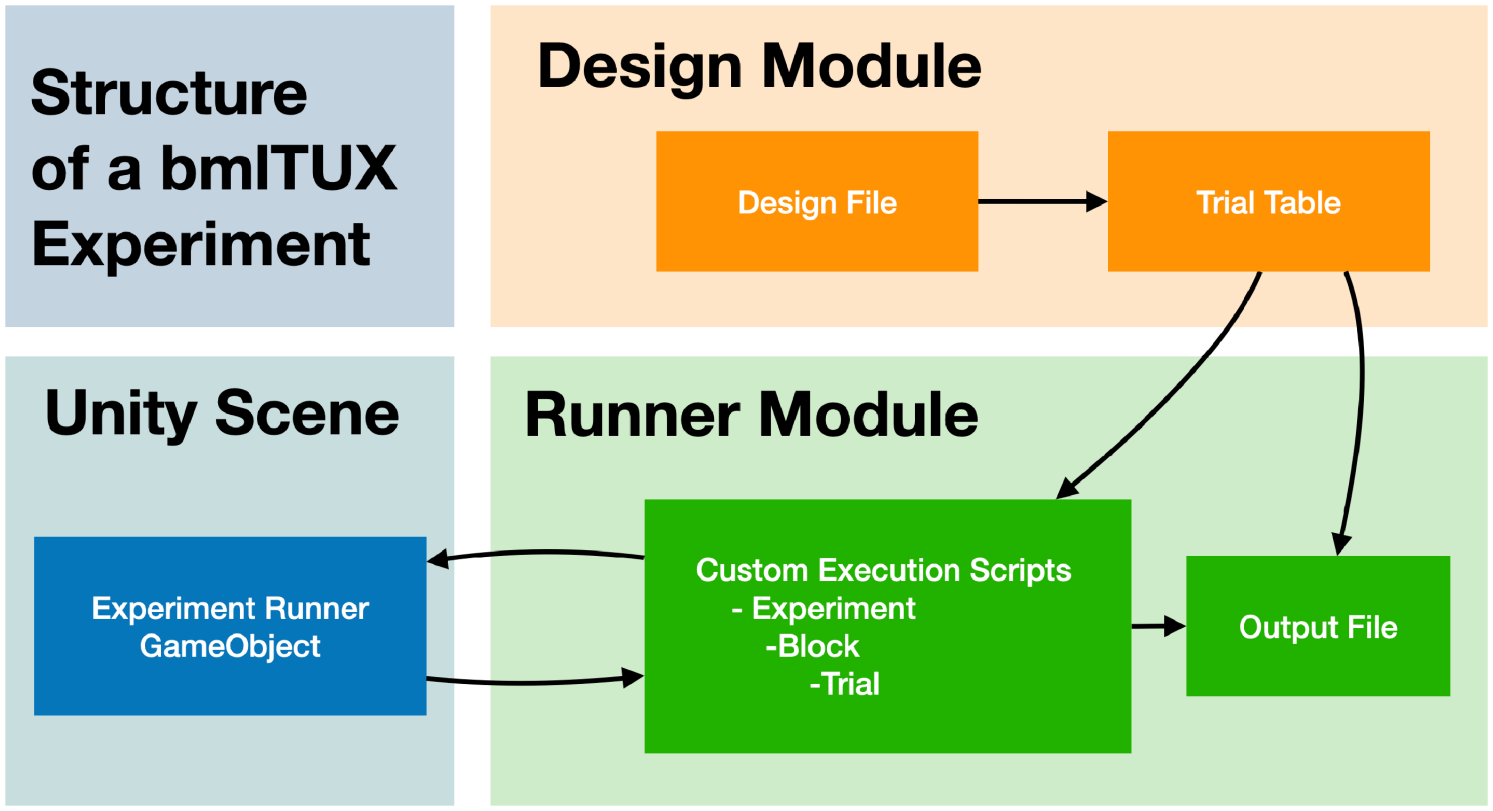
The Structure of a bmlTUX Experiment. Experimental design is configured in a Design File that is used to construct Trial Tables. Upon execution, a Trial Table is passed to the Custom Execution Scripts that are written by the experimenter. These scripts define how the Unity scene is manipulated in each Trial, Block, and Experiment based on the values of the independent variables. After each trial, the output file is updated.

In our implementation, we keep the control of the experimental flow (*Designer* module) entirely separate from the execution of the experiment itself (*Runner* module). The Designer module maintains a *Design file* that contains the specifics of the experimental design. The interface between the two modules is a *Trial Table,* which is based on the design and specifies all trials and the particular order in which they will be executed for each experimental run. Upon execution, the Runner works through the Trial Table and updates it with the values of the dependent variables collected during the experiment (Figure 1).

The Designer is a set of tools that aids with setting up the experiment’s variables and specifying their roles. The Designer stores the configuration of the experiment in a Design File, which is edited using a graphical user interface requiring no coding. The experimenter can declare all factors that control stimuli, the rules by which they are combined into trials, the rules that determine the order in which trials are presented, and a few other parameters that control experimental flow. At this stage, participant variables (e.g., participant age) and dependent variables (e.g., participant responses) can also be specified. When complete, the Design File contains all information required to generate Trial Tables. Since experimental design generally involves random processes (e.g., randomizing the order of trials), a new Trial Table is typically generated for every participant.

The Runner reads a Trial Table line by line and executes the experiment by generating the visual stimulus and logging responses from the participant. While the Designer does not require any programming, the Runner requires short *Custom Execution Scripts* written in C# programming language that turn the variables contained in the Trial Table into the desired visual scene and experimental behavior. Separate custom scripts can be passed to the Runner to customize the behavior of the entire experiment, blocks of trials, and individual trials.

The Design File and Trial Table are stored as editable ASCII files that follow JavaScript Object Notion (JSON) format. Rather than relying on the Designer, they can in principle be created by other means or edited externally and then passed on to the Runner. That way, potential restrictions of the otherwise strictly factorial approach of the Designer can be bypassed.

Creating a simple experiment typically follows the following process:

- Create a scene in Unity.
- Use the included Script Helper Tool to automatically generate a Runner GameObject, a Design File, and all required scripts.
- Configure the Design File with independent and dependent variables and information how to determine the order of trials.
- Customize the Execution Scripts to manipulate the Unity scene as desired. For simple experiments, this only involves editing the Trial script.
- Run the experiment.

## Getting and Using the Toolkit

The toolkit is provided free and open source on GitHub under a Creative Commons Attribution Non-Commercial Share Alike 4.0 International License (http://creativecommons.org/licenses/by-nc-sa/4.0/).

The latest release, comprehensive documentation, and an introductory tutorial can be accessed from https://biomotionlab.github.io/TUX/. Additional information can be found at https://www.biomotionlab.ca/tux/.

## Implementation

### Script Helper Tool

We have included a tool to help set up new experiments. In a new Unity scene, the *Script Helper Tool* can be accessed from the *bmlTUX* menu. This tool can automatically create all required files for a new experiment and will create a Unity GameObject in the scene with the appropriate scripts attached. The Script Helper Tool is not a required part of the toolbox and the advanced users may choose to not use it for the development of experiments. However, it is a very helpful asset for new users, and in the following, we assume that the reader is making use of it.

### The Designer Module

**Figure 2.**
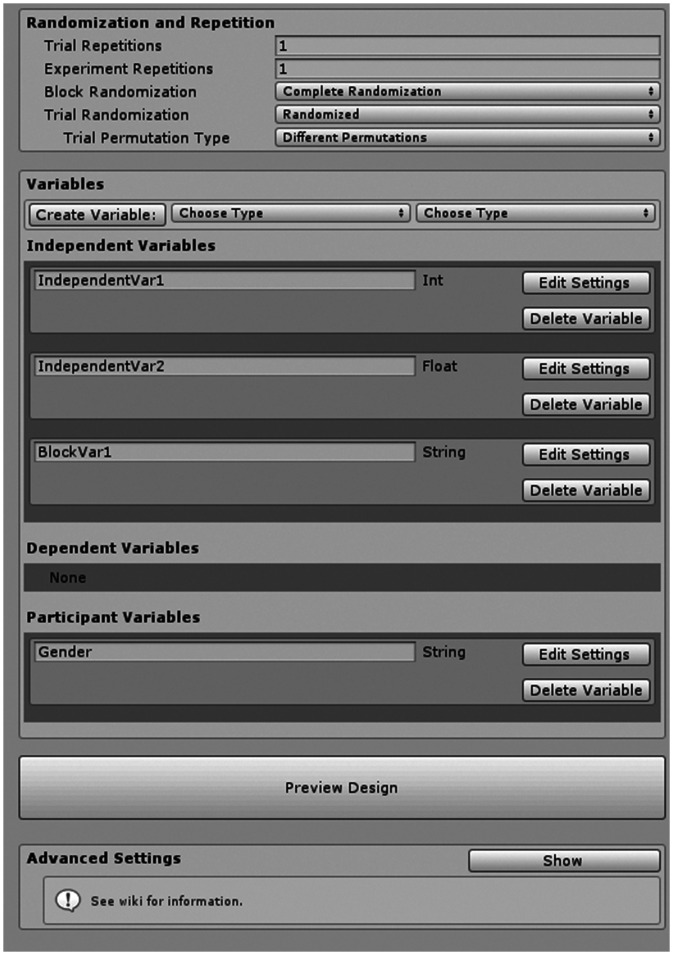
Design File Inspector Interface. Without requiring any code, experimenters can add, remove, and define variables, and adjust randomization, combinatorics, and repetition. An example Trial Table can also be previewed to ensure the design is correctly specified.

To access the Designer, the experimenter has to click on the new Design File created by the Script Helper Tool. This will open a Unity inspector window that allows the creation of variables and configuration of other settings (Figure 2).

#### Defining Variables

The Designer provides a graphical interface within which independent, dependent, and participant variables can be defined, and any counterbalancing, randomization, repetition, and blocking can be configured. Since Unity uses the C# scripting language, which is a statically typed programming language, each variable must be given a data type upon creation. Currently supported types include integer, float (decimal numbers), string (text), and boolean (true/false). There is also support for some Unity classes (Vector3, Vector2, etc.). Unity internally uses the float type for most decimal numbers including time, distances, and rotations, so float variables should be used for variables representing such factors.

#### Independent Variables

Trials are defined by a number of independent variables and the values they can adopt. The Designer enables the experimenter to add new variables using the *Variable Creation* section of the interface. Once created, the experimenter must provide a name and a list of possible values for each variable in the *Values* list. Marking a variable as *Block* affects the order in which trials are run (see trial order section).

#### Combining Variables into Trials

There are several ways by which the toolkit can use to combine the independent variables to form a sequence of trials. Each independent variable can be flagged as *balanced*, *looped, or random*.

*Balanced* variables are added such that each possible value is run at least once. If all variables are set to balanced, then the experiment will be completely counterbalanced such that there will be enough trials to cover every combination of all variable values. For example, a study with 2 variables with 4 levels and 5 levels, respectively, will result in 20 trials (or, if repeated, a multiple thereof) covering every possible combination of values.

*Looped* variables are combined with other variables so that certain values of one variable are tied to certain values of another variable. The toolkit will loop through all values such that there is an equal number of trials with each value. This means that if there are two looped variables, one with *x* possible values and one with *y* possible values, the number of trials will be equal to the lowest common multiple of *x* and *y*. In experiments with both looped variables and balanced variables, all looped variables are added, trials are computed, and then the entire resulting set of looped trials is treated as a single balanced variable for the purposes of counterbalancing.

*Random* variables do not create new trials on their own. If a variable is flagged as random, its value is determined according to a specified probability distribution, but independently of the value of the variable in other trials. If *Even Probability* is selected, all values have an equal likelihood of being selected. If *Custom Probability* is selected, the experimenter can indicate the probability of each value being selected. To make sure that probabilities add up to one, the probability for the last value is calculated automatically. If all independent variables are set to *random* then, by default, the experiment contains only one single trial—unless repetitions are added explicitly (see below).

### Repetitions

By default, the toolkit creates the minimum number of trials required to satisfy the constraints formulated in the experimental design. This set of trials, however, can be repeated several times. To achieve this, the *Trial Repetition* setting can be adjusted. In other cases, experimenters may want to repeat the entire experiment several times. This can be achieved using the *Experiment Repetition* setting.

For clarity, we will distinguish *repetitions* from *blocks*. Blocks are a set of trials grouped by a common value of a block variable. In contrast, repetitions are a set of trials created by duplicating all existing trials. With no block variables, the two *repetition* settings are redundant and behave identically. If the design contains block variables they behave differently (see below).

### Block Order

Due to the different types of repetitions and the existence of blocked variables, there are several order modes available.

- *In Order*: Blocks appear in the order they are created.
- *Custom Order*: Blocks appear in an experimenter-defined order. See online documentation for details.
- *Partial Randomization* (only applicable with repetitions added): Block order is shuffled within repetitions. All Blocks are run before being repeated.

#### Subsettings

- One permutation: A single, random permutation is applied to all repetitions.
- Different permutations (default): Block order is shuffled independently for each repetition.
- *Complete Randomization*: Block order is shuffled, and Experiment Repetitions are shuffled such that they can intermix.

### Trial Order and Randomization

Similar to the block order, the order of the trials within a block can be randomized in different ways:

- *In Order*: Trials appear in the order they are created.
- *Randomized* (with no Block variables): Trial order is shuffled completely, and repetitions can be intermixed with each other.
- *Randomized* (with Block variables): Trial order is shuffled but remains grouped by Block.

#### Subsettings

- *One Permutation*: Trial order is shuffled but will be the exact same for each Block and across repetitions.
- *Different Permutations* (default): Trial order is shuffled differently for each Block and repetition.

During the development of an experiment, it is helpful to set both trial and block randomization to *In Order*. The Trial Table can then be inspected more easily to verify that the intended combinatorics and number of trials are correct.

#### Previewing a Design

Once variables and repetition settings have been configured, a preview of an experimental session can be opened using a button in the Designer Interface. This preview will show a new window displaying an example trial table of the experiment with any randomization applied. This is helpful to preview the number of trials and possible trial orders.

#### Dependent Variables

Dependent variables represent measurements and responses that are collected trial by trial during runtime of the experiment. They are defined in the Designer so that the Runner can update them after each trial and add them to the output file. They can be created in a similar fashion as independent variables and allow the entry of a default value that is used if the experiment fails to record a response.

#### Participant Variables

Participant Variables are variables that do not change over the course of one experimental session. They are typically used to record demographic information from each participant (e.g., age and gender). The toolkit will prompt the experimenter to enter values for the Participant Variables at the start of each experimental session. The allowed possible values can be constrained by checking the *Constrain Values* setting and entering a list of values.

#### Finalizing the Design

Once the configuration of the Designer is complete, the toolkit will create a Trial Table, which specifies the values of all variables for each particular trial and the order in which the trials are executed. Each row of the table is one trial, and each column is one variable. This Trial Table is then passed to the Runner for execution during which it is completed by adding the values of the Dependent Variables.

The toolkit can generate Trial Tables in two ways, *On-The-Fly* (default) or *Pre-Generated*. On-the-fly generation is completely automatic and will likely fulfill the needs for most experiments. In this mode, the toolkit will automatically generate the experiment’s Trial Table from the Design file at runtime.

Although the Design File interface provides all needed controls, both the Design File and Trial Table can be stored and accessed as editable human-readable text files. They can be edited by external tools and then passed to the Runner. This enables randomized variables to be known in advance and allows for fine-tuning of the experimental structure and knowledge of the exact trial order before each session begins. In the advanced settings of the Design File inspector, pregenerated Trial Tables can be created, saved to disk, edited, and then loaded at runtime. In this way, potential restrictions of the otherwise factorial approach of the Designer can be bypassed. For more complex experiments, the built-in Trial Table generation scripts can be bypassed completely. That opens the possibility to generate adaptive designs, for instance, staircase methods.

### The Runner Module

#### Custom Execution Scripts

The Runner Module receives the information specified by the Design File and the resulting Trial File. To turn that information into a running experiment, the experimenter has to provide code that is distributed over four different *Custom Execution Scripts*: The *Runner* script plus three additional scripts that specify the behavior of the experiment on the level of individual trials, blocks of trials, and for the whole experiment.

#### Runner Script

The Runner script is attached to a Unity game object in the scene and references the experiment’s Design File and any other Custom Execution Scripts that are defined. When the Unity scene is run, the Runner script is automatically called and begins the experiment.

### Custom Experiment, Block, Trial Scripts

The script helper tool automatically creates three very basic Custom Execution Scripts that are included with the basic code to run a simple experiment. There are three main hierarchically organized structures that can be customized: *Experiment*, *Block*, and *Trial*. Although the toolkit provides basic scripts for all three categories, most experiments will at minimum require editing the Trial script to describe how the Unity scene should change based on the values of the independent variables in each trial.

**Figure 3.**
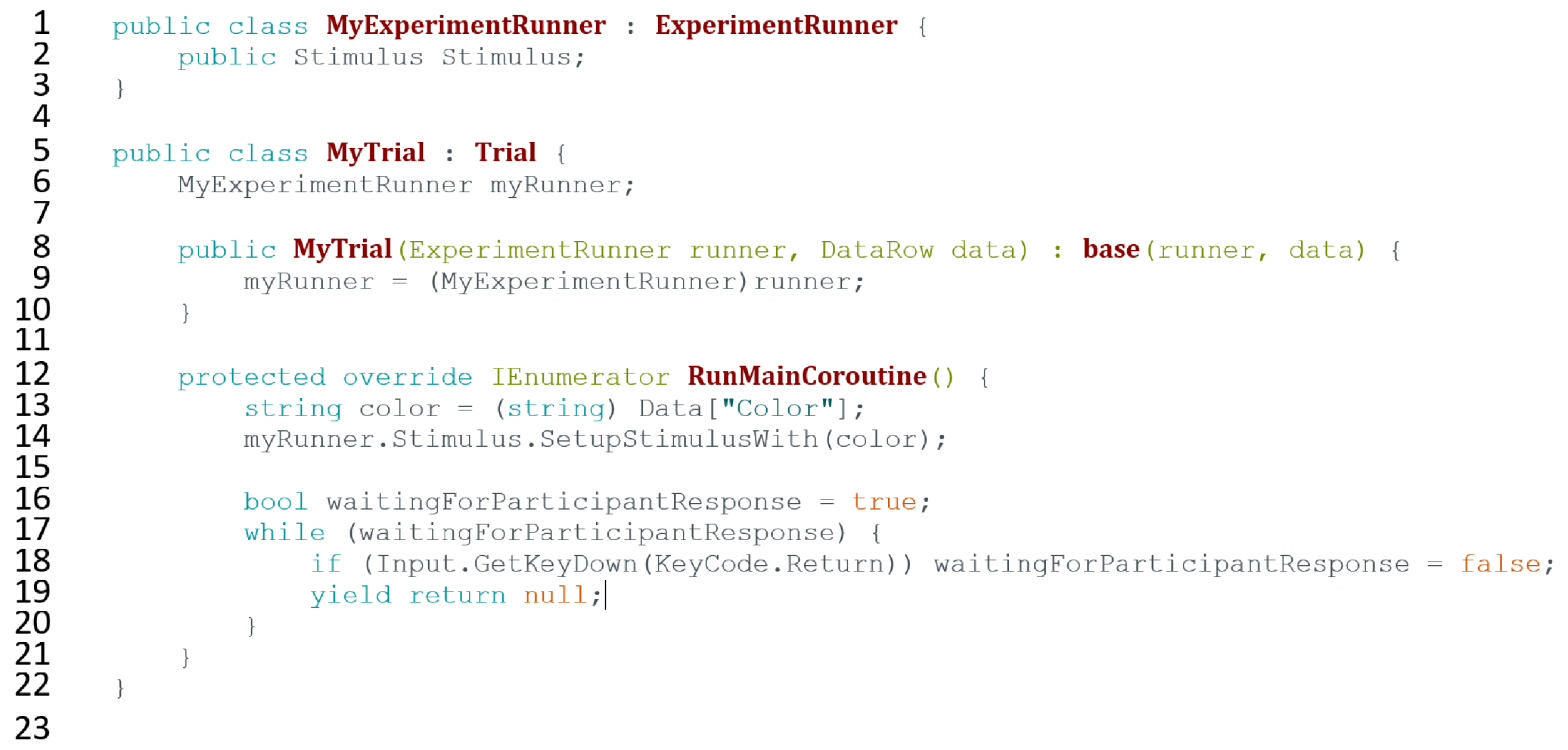
Example Trial Script for a Basic Experiment. Note that each trial shows a stimulus of varying colors (L 13-14), and then waits for the user to hit the return key (L 16-20). It creates a subclass of the class Trial that is included in bmlTUX (L 5-10), which already contains all the code for a simple trial to run. This script adds custom behavior to a trial’s main execution function, RunMainCoroutine (L 12). It overrides (customizes) the function such that each trial will execute this custom behavior rather than the built-in code. In a separate script, the custom ExperimentRunner script attached to a GameObject in the Unity scene stores a reference to the Stimulus object (L 1-3). This is all the code needed to run this simple experiment.

Each Custom Execution Script extends a C# class defined by the toolkit. These classes (Trial, Block, and Experiment) all share a series of automatically called functions where custom behavior can be added. Each script can be extended through three types of such functions: (a) *Preexecution* functions are useful for initialization, instructions, and setup; (b) *Postexecution* functions are useful for cleanup, measurement, and recording responses; and (c) the *Main-execution* function. The Main-execution function can only be customized for Trials, since for Experiments and Blocks, the execution simply consists of running trials. For very simple experiments, the only coding required is writing a custom Main-execution function (Figure 3).

The toolkit automatically provides the values of independent variables to the Trial and Block scripts. Functions can then use these values to appropriately modify the Unity scene for each trial.

More detailed information about writing custom scripts can be found in the official documentation and tutorials. Note that these custom extension scripts can reference and call objects and functions from any other C# scripts, allowing for more advanced customizations and experimental structures to be coded.

#### Running an Experiment

**Figure 4.**
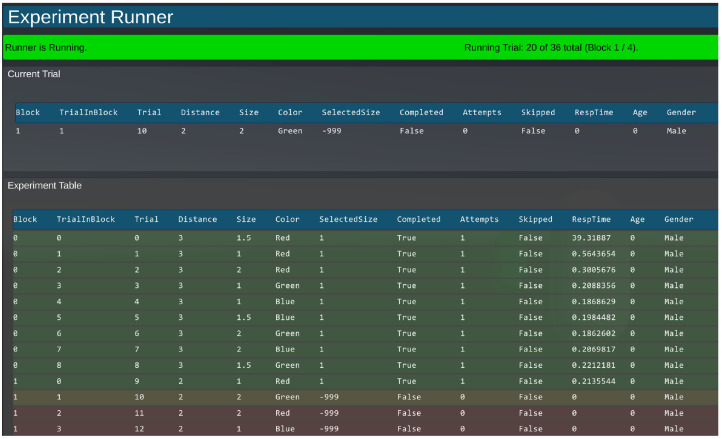
Runner Interface While Running an Experiment. Current progress through trials and blocks is shown, and measured variables are updated as trials are completed. This interface can be shown on a secondary display if desired.

Once the Designer has been configured and the Runner has been provided with the required scripts, the experiment can be run within the Unity Editor or as a built executable program. When run, the toolkit displays a window showing the Experiment Runner Interface and starts a new experimental session. The interface prompts the experimenter for the location to save the output file, entry of participant variables, and other settings for starting an experimental session. The interface can also provide some additional functionality including the ability to watch the progress of the experiment in real time, and the ability to skip and navigate between trials (Figure 4). When the *Start Experiment* button is pressed, the toolkit executes the experiment and runs through all trials. An output file is automatically created, which is basically a copy of the Trial Table with added columns that contain the values of the dependent variable(s).

## Discussion

Using bmlTUX, an experimenter must write much less code for simple and moderately complicated experiments than would be required using UXF or USE, since it automatically handles the code for configuring variables, counterbalancing, creating the trials, ordering trials, and so on. Although the toolkit simplifies this process greatly, it remains highly customizable and flexible even for very advanced experimental designs. The toolkit drastically reduces the time needed to get an experiment up and running and has powerful features for modifying the experimental design incrementally without hassle. This makes experimental creation in Unity much more approachable to novice coders and much faster for expert coders, particularly in situations where frequent iterative design is desired.bmlTUX enables quickly designing and running experiments in Unity Game Engine, which frees more time to be spent on increasing realism and graphics. Combined with its built-in compatibility with VR frameworks, bmlTUX is an especially useful tool for creating more *naturalistic* vision experiments in virtual environments. Such naturalistic experiments allow experimenters to present realistic visual environments while maintaining a high degree of experimental control (Scarfe & Glennerster 2019).bmlTUX can store and swap multiple variants of its design with ease using a drag-and-drop interface. This enables experimenters to have multiple working versions of an experiment within the same project that can be interchanged at a moment’s notice. This is especially useful for keeping pared-down versions of experiments for debugging, pilot testing, or training purposes. It is also useful for storing a history of changes to the experimental design over time.

The toolkit is designed to be approachable by novice-level coders. The interface for configuring the experiment’s variables, the experiment controls, and the automatic creation of trials and blocks is excellent for minimizing the time required to build an experiment, even for experimenters with little coding knowledge. We have pilot-tested the use of the toolkit on several small groups of undergraduate and graduate students with novice-level coding and Unity knowledge, and all were able to design simple experiments after only a few hours of use. We have provided a basic tutorial to help get started and learn the fundamentals of working with the toolkit and have created a wiki page with more detailed documentation of the more advanced features and customizations. Despite being targeted for novice coders, advanced coders will benefit from using bmlTUX due to its flexibility, customizability, fast implementation, and iterative capabilities.

Despite the flexibility and ease of use of the toolkit, it does have some limitations. For example, USE has much more precise control and measurement of timing events and has built-in tools for helping with integration of external hardware compared with bmlTUX. For research where high-precision timing is important, the USE framework might be more suitable since bmlTUX’s timing accuracy is currently limited to the update rate of the Unity program (usually around 60–120 Hz). In addition, the toolkit is somewhat restricted to factorial designs, whereas USE is more flexible through its Control Structure interface. We made this decision consciously to target a more coding-averse audience, since it simplifies creating custom scripts. However, it means that it may be difficult to implement more complicated procedures such as adaptive staircase designs. Furthermore, although hidden *behind the scenes*, bmlTUX uses loops and coroutines in addition to event systems to control the flow between trials and blocks, whereas UXF and USE rely exclusively on event systems. Although this improves the learning curve for novice coders, advanced coders may find this slightly less expandable for complex projects. There are plans to allow custom scripts to access internal events in a future release.

bmlTUX provides an approachable and powerful set of tools to create experiments with the Unity Game Engine and caters to both novice and advanced coders. The toolkit simplifies the process of getting experiments up and running quickly without the hassle of complicated scripting.
